# A Patient with Diabetes Mellitus and Hypertension Presenting with Left Flank Pain and Hypotension

**DOI:** 10.34067/KID.0000000962

**Published:** 2026-02-26

**Authors:** Shubham Dubey, Kapil Sejpal, Manish Balwani

**Affiliations:** 1 Department of Nephrology, Datta Meghe Institute of Higher Education and Research, Wardha, India; 2 Department of Nephrology, Saraswati Kidney Care Center, Nagpur, India

**Keywords:** AKI, anemia, arteries, clinical nephrology, imaging, interventional nephrology, kidney anatomy, renal carcinoma, renal failure, vascular

## Abstract

**Figure:**
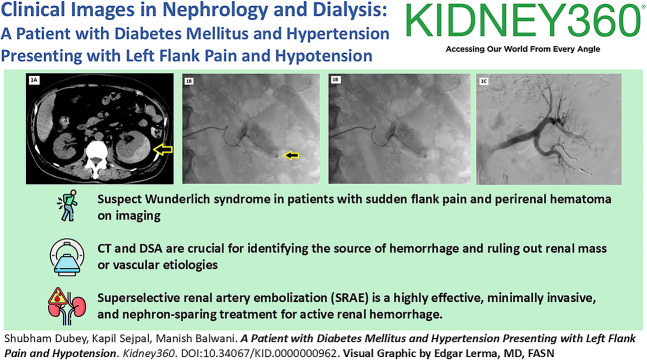

## Case Description

**Figure 1:**
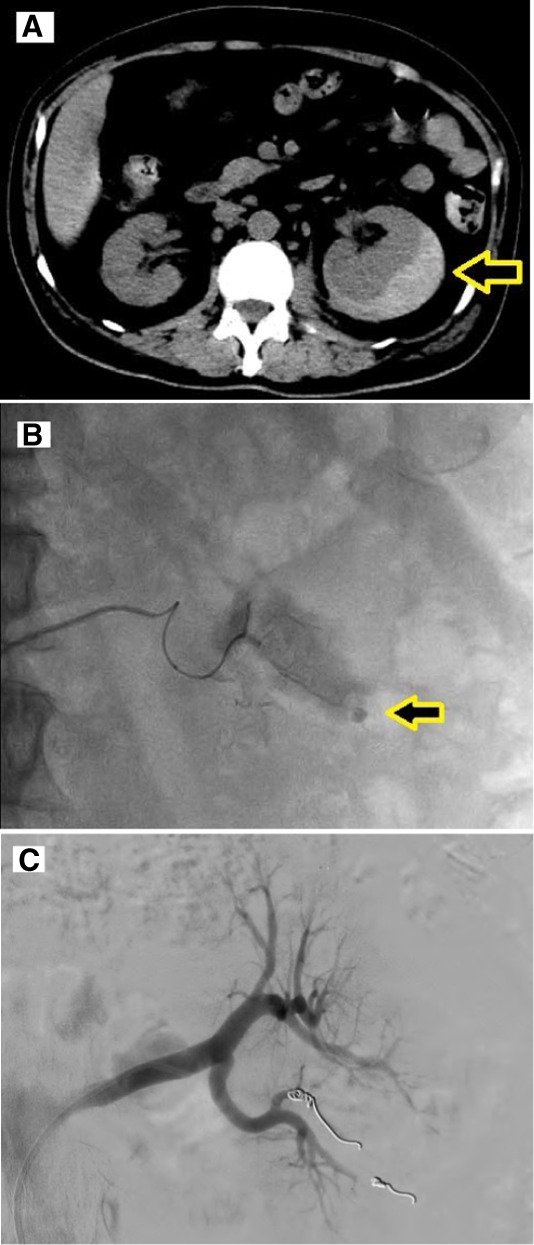
**Radiological confirmation and embolization of spontaneous renal hemorrhage.** (A) Noncontrast CT showing a crescent-shaped left-sided subcapsular perirenal hematoma (arrow). (B) DSA showing focal contrast extravasation from a lower pole segmental artery (arrow). (C) Postembolization angiogram showing successful occlusion of the segmental artery.

A 64-year-old man with a history of type 2 diabetes and hypertension presented with sudden onset of left-sided flank pain, fever, and vomiting for 1 day. On examination, he had tachycardia (heart rate 102), hypotension (BP 100/70 mm Hg), and a temperature of 37.6°C. There was moderate tenderness in the left flank and a palpable mass on palpation. Laboratory investigations revealed an elevated white blood cell count (16,200 cells/μl), low hemoglobin level (9.4 g/dl), high BUN level (116 mg/dl), and serum creatinine level of 3.4 mg/dl. Renal ultrasonography revealed a crescent-shaped hypodense collection around the left kidney. Noncontrast computed tomography (CT) confirmed a left-sided subcapsular perirenal hematoma with a maximum thickness of 3 cm (Figure 1A). As there was no history of trauma, further tests were performed to identify other causes. CT renal angiography ruled out a renal mass and vasculitis. Autoimmune tests were negative, and clinical exome sequencing did not reveal any connective tissue disorders. Owing to ongoing symptoms and imaging suggestive of active bleeding, digital subtraction angiography (DSA) was performed, which showed a focal contrast leak from a segmental branch of the left renal artery, indicating an active hemorrhage (Figure 1B). Superselective renal artery embolization (SRAE) was performed using two coils (3×14 and 3×5 mm). Postembolization angiography confirmed successful closure of the bleeding vessel (Figure 1C). During his hospital stay, the patient received four units of packed red blood cells, along with supportive care and antibiotics. The patient's hemoglobin level and renal function improved, and he was discharged in stable condition with instructions for close follow-up.

## Discussion

Wunderlich syndrome is a rare entity characterized by spontaneous hemorrhage in the subcapsular or perirenal space, without any evidence of trauma.^1^ It typically presents with Lenk triad of sudden flank pain, a palpable mass, and signs of shock.^2^ The common causes include angiomyolipoma, renal cell carcinoma, rupture of renal artery aneurysm, and vasculitis syndromes.^3^ In this case, no cause was found after extensive workup, making it an idiopathic or spontaneous Wunderlich syndrome.

Noncontrast CT is the primary imaging modality, often showing a perirenal hematoma. For stable patients, CT angiography helps rule out mass or vascular lesions. DSA remains the gold standard for diagnosis and treatment through SRAE, which preserves the renal parenchyma.^4^ Management includes conservative treatment initially, followed by renal angiography to look for active bleeding. If the bleeding is severe, leading to page kidney, the patients might require nephrectomy.^5^ Prompt and accurate diagnosis of the underlying cause is necessary to prevent morbidity and mortality in such patients.

## Teaching Points

- Suspect Wunderlich syndrome in patients with sudden flank pain and perirenal hematoma on imaging.
- CT and DSA are crucial for identifying the source of hemorrhage and ruling out renal mass or vascular etiologies.
- SRAE is a highly effective, minimally invasive, and nephron-sparing treatment for active renal hemorrhage.
